# Behavioral response of *Panonychus citri* (McGregor) (Acari: Tetranychidae) to synthetic chemicals and oils

**DOI:** 10.7717/peerj.10899

**Published:** 2021-04-05

**Authors:** Muhammad Asif Qayyoum, Zi-Wei Song, Bao-Xin Zhang, Dun-Song Li, Bilal Saeed Khan

**Affiliations:** 1 Guangdong Provincial Key Laboratory of High Technology for Plant Protection/Plant Protection Research Institute, Guangdong Academy of Agricultural Sciences, Guangzhou City, Guangdong, China; 2 Department of Plant Protection, Ghazi University, Dera Ghazi Khan, Dera Ghazi Khan, Punjab, Pakistan; 3 Department of Entomology, University of Agriculture Faisalabad, Faisalabad, Punjab, Pakistan

**Keywords:** Leaf surfaces, Lethal concentration, Sub-lethal concentration, Dispersal pattern, Colonization

## Abstract

**Background:**

*Panonychus citri* (McGregor) (Acari: Tetranychidae) population outbreaks after the citrus plantation’s chemical application is a common observation. Dispersal behavior is an essential tool to understand the secondary outbreak of *P. citri* population. Therefore, in the current study, the dispersal activity of *P. citri* was observed on the leaf surfaces of *Citrus reticulata* (Rutaceae) treated with SYP-9625, abamectin, vegetable oil, and EnSpray 99.

**Method:**

Mites were released on the first (apex) leaf of the plant (adaxial surface) and data were recorded after 24 h. The treated, untreated, and half-treated data were analyzed by combining the leaf surfaces (adaxial right, adaxial left, abaxial right, and abaxial left). All experiments were performed in open-air environmental conditions.

**Results:**

The maximum number of mites was captured on the un-treated or half-treated surfaces due to chemicals repellency. Chemical bioassays of the free-choice test showed that all treatments significantly increased the mortality of *P. citri* depending on application method and concentration. A significant number of mites repelled away from treated surfaces and within treated surfaces except adaxial left and abaxial right surfaces at LC_30_. In the no-choice test, SYP-9625 gave maximum mortality and dispersal by oils than others. No significant differences were observed within the adaxial and abaxial except abaxial surface at LC_30_. Therefore, the presence of tested acaricides interferes with *P. citri* dispersal within leaf surfaces of plantations depending on the mites released point and a preferred site for feeding.

## Introduction

The citrus red mite, *Panonychus citri*, is a serious pest of the citrus growing region all over the world (Gotoh & Kubota, 1997; Kasap, 2009; Faez et al., 2018b; Korhayli, Barbar & Aslan, 2018) as well as in China (Yuan et al., 2010; Fang et al., 2013; Liu et al., 2019). The immature and adult stages feed on leaves and fruit by giving stippling damage, which inhibits the photosynthesis process and leads shoot dieback and leaf/fruit dropping (Kranz, Schmutterer & Koch, 1978). Server infestation in the field may cause irritation and allergic reactions to citrus workers (Fernández-Caldas et al., 2014).

The chemicals application is a preferred method to control *P. citri* by the farmers in citrus orchards (Gotoh & Kubota, 1997; Chen et al., 2009; Kasap, 2009; Fadamiro et al., 2013; Faez et al., 2018a, 2018b; Karmakar, 2019; Liu et al., 2019). SYP-9625 and abamectin are commonly used among synthetic chemicals against citrus pests in China (Gu et al., 2010; Hu et al., 2010; Liu et al., 2018; Chen et al., 2019). It is essential to find alternative products (Isman, 2008; Tak & Isman, 2017) for synthetic chemicals due to serious threats to non-target organisms and the environment (Kumral, Cobanoglu & Yalcin, 2010; Chen & Dai, 2015). Agricultural mineral oils (EnSpray 99) are compatible with predatory mites application and effective against horticultural crop pests (Wang, Gu & Bei, 2004; Chen & Zhan, 2007; Xue et al., 2009a, 2009b; Zhou et al., 2011; Zhuang et al., 2015). Vegetable oils are also considered an alternative due to toxicity and repellency against target pests (Koulbanis et al., 1984; Ismail et al., 2011; Oliveira et al., 2017). Vegetable oil extracted from kitchen/household waste (vegetable remaining) were used in this study. Institute of Zoology, Guangdong Academy of Sciences, China, provided this kitchen vegetable waste oil (as a trial product).

Environmental contamination such as pesticides can influence mites behavioral activities on leaves or plants (Ibrahim & Yee, 2009; Lima et al., 2013; Cordeiro, Corre & Guedes, 2014; Monteiro et al., 2019a). The behavioral changes due to chemicals affect pest management strategies (Guedes et al., 2016). The population outbreaks of plant-feeding mites after the chemical application on the horticultural crops are very common (AIiNiazee & Cranham, 1980; Zwick & Field, 1987). The abrupt increase of the mites population has many suggestions by the researchers; the most critical explanation suggests the impact of chemicals on the natural enemies (AIiNiazee & Cranham, 1980; Dittrich et al., 1980; Zwick & Field, 1987). Iftner & Hall (1983) reported that increasing the chemical application rates in the absence of natural enemies also increases pest numbers. Since, the impact of agrochemicals on target pest or insect can be assessed through the application rate (lethal and sublethal), application timing, and mode of action. The use of the sublethal effect of chemicals is considered a more accurate approach to measure toxicity, which changes individuals behavioral responses that survive from toxic exposure (Desneux, Decourtye & Delpuech, 2007; Biondi et al., 2013; Turchen et al., 2016; Alves, Casarin & Omoto, 2018).

Dispersal behaviors define as any movement from one place to another for the survival of any organism due to environmental stress or non-viable to live (e.g., lack of food or surrounding climatic constraints) (Clobert et al., 2001; Ims & Hjermann, 2001). Dispersal movement done in three stages; emigration, a vagrant stage, and immigration (Ronce, 2007), which depend on the species life cycle, sex, environmental variations, space, and time (Dunning et al., 1995; Hanski, 1998, 1999; Turchin, 1998; Bergman, Schaefer & Luttich, 2000; Bowler & Benton, 2005).

The dispersal behavior of mites uses active or passive dispersal mechanisms (Evans, 1992; Sabelis & Afman, 1994; Tixier et al., 1998; Perotti & Braig, 2009). Active dispersal (walking) is the most preferred mechanism in mites due to morphological characteristics and short-range travel (Strong, Slone & Croft, 1999; Melo et al., 2014; Monteiro et al., 2019a). Like most of the tetranychids, *Panonychus citri* also do passive dispersal by silk threads (aerial dispersal) to overcome crowding, food depletion (Bell et al., 2005), and light-dependent (Pralavorio, Fournier & Millot, 1989). In this study, we evaluated the lethal and sublethal effects of selected pesticides on the dispersal pattern of *P. citri* by treating the leaf surfaces. We hypothesized that *P. citri* response towards chemicals treatment may be a reason for the population outbreak in the field conditions.

## Materials and Methods

### Mite culture

Mite culture was regularly maintained since 2019, on lemon leaves with the water-saturated sponge. The culture was reared in the growth chamber with a 16:8h (Light:Dark) photoperiod and 26 ± 1 °C temperature. One to three-day-old adult females (He et al., 2011; Alves, Casarin & Omoto, 2018) were used for said experimentation reared in the laboratory for several generations (more than 50 generations). The 1–3-day-old adult females were used due to fully developed adultery and ready for egg-laying after 4–5 days. The mite culture was shifted to the open-air environment 1 month before the experiment to acclimatize.

### Plants

Citrus plants (*Citrus reticulata*) approximately 1–2 months old were used after shifting to the pots. The plants with 7–8 leaves were used by leaving six leaves (3 on the right and left side) and cutting them. All plants were washed three times with water to be sure not to have any arthropods on them. The bottom of each plant stem was wrapped with wet tissue paper and maintained wet to keep mites on the plant. All plants were manured and watered accordingly under reasonable conditions during January.

### Chemicals

SYP-9625 30% EC and Abamectin 5% EC, EnSpray 99% EC (EnSpray 99), and vegetable oil 99% were used in this research. Chemicals and EnSpray were bought from the local market. The degummed vegetable oil was extracted from household daily kitchen vegetable waste that was provided by the Institute of Zoology, Guangdong Academy of Sciences.

Each chemical toxicity was calculated using a modified leaf dip bioassay (Wang et al., 1971; Nauen, 2005) previously in the laboratory. The selection concentrations of each chemical were made with 10–90% corrected mortality after 24 h. Lethal and sublethal concentrations of each chemical were calculated by probit analysis using SPSS version 22.0 software (Weinberg & Abramowitz, 2016). In this experiment, we used LC_30_ (0.065%, 0.049%, 0.024% and 0.08%) and LC_50_ (0.196%, 0.110%, 0.051% and 0.024%) for SYP-9625, Abamectin, Vegetable oil and EnSpray 99, respectively.

### Experimental methodology

**Figure 1:**
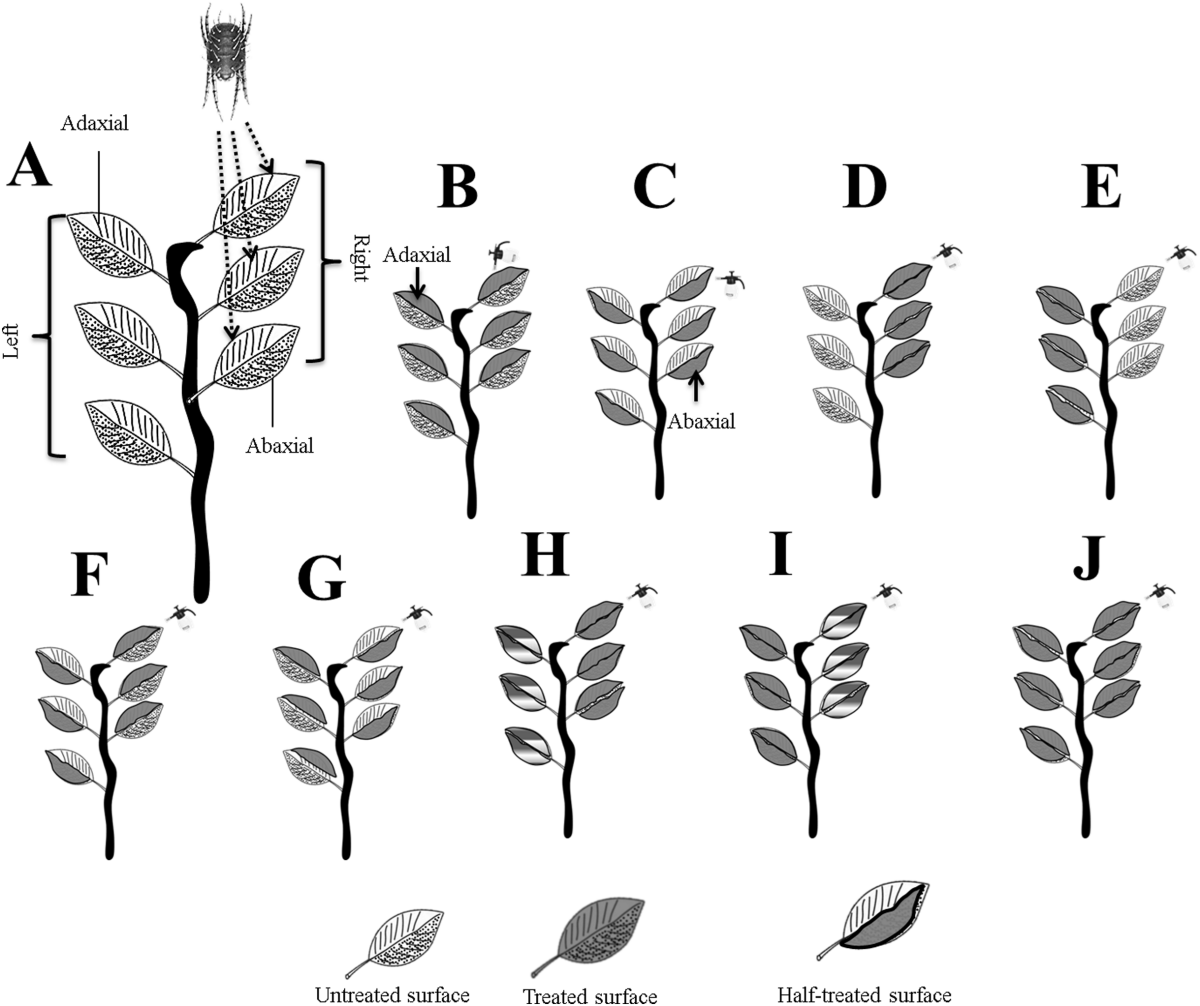
Systematic outline of the experimental layout. (A) Mites were released on the right adaxial (ADR) surfaces; (B) ADR and ADL; (C) ABR and ABL; (D) ADR and ABR; (E) ADL and ABL; (F) ADR and ABL; (G) ABR and ADL; (H) for full treated ADR and ABR, and for half treated, ADL and ABL; (I) for full treated ADL and ABL, and for half treated ADR and ABR, and (J) whole plant treated. Letters were assigned to leaves surfaces as; adaxial right (ADR), adaxial left (ADL), abaxial right (ABR), and abaxial left (ABL). Photo credit: Muhammad Asif Qayyoum.

The method adopted by Iftner & Hall (1983) was followed for the current experiment. Letters were assigned to leaves surfaces as; adaxial right (ADR), adaxial left (ADL), abaxial right (ABR), and abaxial left (ABL). We used a free choice and no choice method by dividing it into nine small experiments, as shown in Fig. 1. Chemicals were applied to the treated leaf surface with a hand sprayer. Untreated part of leaflet or surfaces was protected from chemicals spraying by cardboard shield and plastic bags. Each plant’s ground surface was covered with plastic with double side sticky tape on the edge. The right adaxial surface was selected for easy to release mites (20 mites × 3 surfaces) and identified mites location from the inoculated surface after 30 min of chemicals application. Mites were captured 24 h by location as per the experimental layout. The mites on the leaf surface, wet tissue paper (chemically treated), and plastic cover (chemical sprayed) were considered as dead. The mites not found as live or dead were considered missing mites. The experiments were used with three replications.

The treated, un-treated, and half-treated data were combined for leaf surfaces (ADR, ADL, ABAR, and ABL) further analysis. All experiments were performed in open-air environmental conditions.

### Statistical analysis

The mean number of mites (LC_30_ vs LC_50_, Treated vs Un-treated, Treated vs Half-treated, and Adaxial vs Abaxial) were analyzed using an independent sample *t*-test. The difference between control and treatments captured mites means were analyzed using the general linear model (GLM) for ANOVA with Tukey’s HSD test (*P* < 0.05). All statistical analysis procedures were calculated with Minitab^®^ 17.3.1 version (Minitab, 2016). Graphical representation was done using GraphPad prism^®^(Motulsky, 2007) and OriginPro (Edwards, 2002).

A correlation analysis was conducted by comparing toxicity (% mortality) and % mites present on treated, un-treated, and half-treated surfaces to better understand the relationship between the behavioral responses of *P. citri*. Pearson correlation (*r*) and calculating *t* distribution value formulas were used in R.

$$r=\;\frac{\sum (x-mx)(y-my)}{\sqrt{\sum {\left(x-mx\right)}^{2}\sum {\left(y-my\right)}^{2}}}$$

$$t=\;\frac{r}{\sqrt{1-\;r^{2}}}\sqrt{n-2}$$

***n*** is the length of factor (df = n − 2) in two vectors (***x*** (toxicity) and ***y*** (mites observed on treated or untreated or half treated surfaces)) while ***mx*** and ***my*** are the means of vectors. The significant level can be determined by the *t*-value.

## Results

### Toxicity

Compared to control, acute toxicity of treatments was found significantly different within each dose in all experiments except in exp. no. 8 at LC_30_. There was significant difference between doses within abamectin (For exp. no. 3; *t*_−5.56_ = −5.00; *P* = 0.007), SYP-9625 (For exp. no. 4; *t*_−7.78_ = −3.50; *P* = 0.025) and EnSpray (For exp. no. 4; *t*_−8.89_ = −8; *P* = 0.001, For exp. no. 6; *t*_−6.67_ = −3.464; *P* = 0.026) and vegetable oil (For exp. no. 8; *t*_−4.44_ = −2.828; *P* = 0.047) than others. Differences in toxicity (from LC_30_ to LC_50_) of chemicals to adult (female) *P. citri* occurred among experiments depending on application methods, with ranges in SYP-9625, abamectin, vegetable oil, and EnSpray of 1.156–2.399-fold, 1.33–5.556 fold, 1.249–5.005 fold and 0–8.889 fold, respectively. Maximum toxicity (%) was observed in the no-choice experiment (the whole plant treated—exp. no. 9), and SYP-9625 more toxic (except in exp. no. 2) than others in all experiments (Table 1).

**Table 1:** Toxicity of *Panonychus citri* (% mortality ± SE) 24 h within nine experiment combinations.

Treatment	Concentrations (%)	Experiments								
		1	2	3	4	5	6	7	8	9
SYP-9625	LC_30_	10 ± 0 A	0 ± 0 B	8.889 ± 1.11 A	5.556 ± 1.11 A	6.667 ± 0 A	5.556 ± 2.22 A	13.33 ± 1.925 A	6.667 ± 0 A	35.556 ± 5.556 A
	LC_50_	12.223 ± 2.22 a	2.22 ± 1.11 b	12.22 ± 1.11 a	13.33 ± 1.925 a	8.889 ± 2.22 a	7.778 ± 1.11 a	17.778 ± 1.11 a	7.778 ± 1.11 a	41.11 ± 4.44 a
Abamectin	LC_30_	2.22 ± 1.11 BC	2.22 ± 1.11 B	0 ± 0 B	2.22 ± 1.11 B	7.778 ± 1.11 A	3.33 ± 0 A	10 ± 0 A	3.33 ± 1.925 A	12.22 ± 1.11 B
	LC_50_	6.667 ± 0 ab	3.33 ± 0 b	5.556 ± 1.111 b	6.667 ± 0 abc	12.22 ± 2.22 a	4.44 ± 1.11 ab	16.667 ± 1.925 a	4.44 ± 1.11 ab	22.22 ± 4.006 b
Vegetable oil	LC_30_	7.778 ± 1.11 AB	0 ± 0 B	0 ± 0 B	0 ± 0 B	3.33 ± 0 A	2.22 ± 2.22 A	6.667 ± 1.925 AB	1.11 ± 1.11 A	8.889 ± 1.11 B
	LC_50_	13.33 ± 1.925 a	2.22 ± 1.11 b	2.222 ± 1.111 bc	3.33 ± 1.925 bc	5.556 ± 1.11 ab	6.667 ± 0 a	12.22 ± 1.11 a	5.556 ± 1.11 ab	11.11 ± 1.11 bc
EnSpray 99	LC_30_	12.22 ± 1.11 A	7.778 ± 1.11 A	0 ± 0 B	0 ± 0 B	4.444 ± 1.11 A	0 ± 0 A	8.889 ± 2.22 A	4.44 ± 2.22 A	7.778 ± 1.11 B
	LC_50_	12.22 ± 2.94 a	8.889 ± 1.11 a	0 ± 0 c	8.889 ± 1.11 ab	7.778 ± 1.11 a	6.667 ± 1.925 a	13.33 ± 3.849 a	5.556 ± 1.11 ab	7.778 ± 1.11 c
Control		0 ± 0 B, b	0 ± 0 B, b	0 ± 0 B, c	0 ± 0 B, c	0 ± 0 B, b	0 ± 0 A, b	0 ± 0 B, b	0 ± 0 A, b	0 ± 0 B, c
Statistics at df = 4,14	LC_30_	*F* = 18.08	*F* = 23	*F* = 64	*F* = 12	*F* = 12.17	*F* = 2.05	*F* = 7.69	*F* = 3.33	*F* = 22.79
		*P* = 0.000	*P* = 0.000	*P* = 0.000	*P* = 0.001	*P* = 0.001	*P* = 0.164	*P* = 0.004	*P* = 0.056	*P* = 0.000
	LC_50_	*F* = 7.50	*F* = 7.50	*F* = 35.50	*F* = 10.60	*F* = 8.35	*F* = 4.88	*F* = 11.97	*F* = 5.83	*F* = 33.18
		*P* = 0.005	*P* = 0.005	*P* = 0.000	*P* = 0.001	*P* = 0.003	*P* = 0.019	*P* = 0.001	*P* = 0.011	*P* = 0.000

**Note:**

Capital letters indicate the differences among the LC_30_ of treatments with control and lowercase letters indicates differences among the LC_50_ of treatments with a control. Different letters in the same column are significantly different at the Tukey test (α = 0.05).

### Re-captured *Panonychus citri*

According to Fig. 1, the experimental layout is further divided into three parts; Treated vs Untreated (Experiments 1–6), Treated vs Half-treated (Experiments 7–8), and the whole plant treated (Adaxial vs Abaxial) (Experiment 9).

**Figure 2:**
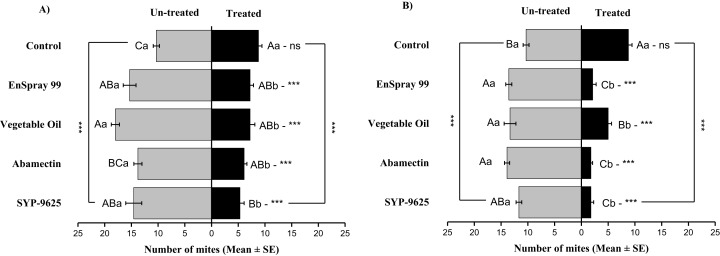
The number of *Panonychus citri* (Mean ± SE) re-captured after 24 h on the adaxial surface of leaves (right side); (A) LC_30_, (B) LC_50_. A significant difference was observed between treatments than control within treated (df = 4,14; For LC_30_: *F* = 3.37, *P* = 0.018; for LC_50_: *F* = 28.01, *P* = 0.000) and untreated surfaces (For LC_30_: *F* = 7.41, *P* = 0.000; for LC_50_: *F* = 4.74, *P* = 0.003). The capital letters indicate differences among the treatments (Treated or Un-Treated); lowercase letters indicates differences between treated and untreated surfaces, Tukey test (α = 0.05). Significant difference “***” and non-significant difference “ns”.

Mites dispersal within the ADR surface were observed 40–82.24% (LC_30_) and 53.7–94.067% (LC_50_) from treated to untreated. The difference between treated and untreated was significantly recognizable. A significant difference was observed in all treatments between the mean number of mites captured on the treated and un-treated on the ADR: SYP-9625 (*t*_−9.22_ = −5.56; *P* = 0.000), Abamectin (*t*_−7.667_ = −8.37; *P* = 0.000), vegetable oil (*t*_−10.78_ = −9.17; *P* = 0.000) and EnSpray 99 (*t*_−8.11_ = −5.78; *P* = 0.000) except control (*t*_−8.11_ = −5.78; *P* = 0.097), at LC_30_ while similar results found by applying the LC_50_ doses. The number of mites captured on the treated ADR surface was lower than the number of mites captured on the untreated surface. A maximum number of mites were observed under the un-treated ADR surface at LC_30_ dose of vegetable oil than in the others (Fig. 2).

**Figure 3:**
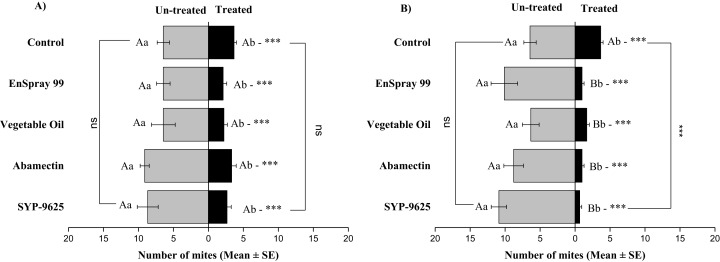
The number of *Panonychus citri* (Mean ± SE) re-captured after 24 h on the adaxial surface of leaves (left side); (A) LC_30_, (B) LC_50_. A significant difference was observed between treatments than control within treated at LC_50_ (*F* = 14.67, *P* = 0.000). The capital letters indicate differences among the treatments (Treated or Un-Treated); lowercase letters indicates differences between treated and untreated surfaces, Tukey test (α = 0.05). Significant difference “***” and non-significant difference “ns”.

On the Adaxial surface of left side (ADL), a significant difference was observed within all treatments between the mites captured on the treated and un-treated ADL surfaces: control (*t*_−2.778_ = −2.94; *P* = 0.015 and *t*_−2.778_ = −2.94; *P* = 0.015), SYP-9625 (*t*_−6_ = −3.45; *P* = 0.006 and *t*_−10.22_ = −8.72; *P* = 0.000), Abamectin (*t*_−5.778_ = −6.28; *P* = 0.000 and *t*_−7.78_ = −5.29; *P* = 0.001), vegetable oil (*t*_−4.22_ = −2.37; *P* = 0.042 and *t*_−4.67_ = −3.78; *P* = 0.004) and EnSpray 99 (*t*_−4.33_ = −3.99; *P* = 0.002 and *t*_−9.11_ = −4.77; *P* = 0.001) on the LC_30_ and LC_50_ doses respectively. A higher number of mites captured on the un-treated surface at LC_50_ of SYP-9625 than others (Fig. 3).

**Figure 4:**
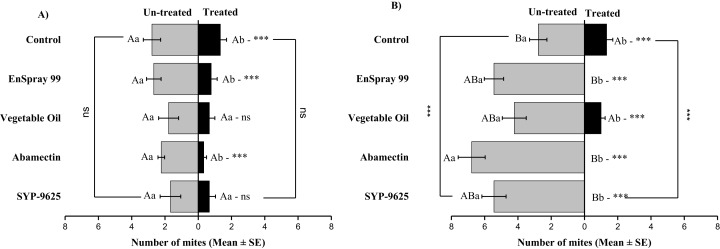
The number of *Panonychus citri* (Mean ± SE) re-captured after 24 h on the abaxial surface of leaves (right side); (A) LC_30_, (B) LC_50_. A significant difference was observed between treatments than control at LC_50_ (For treated: *F* = 10.86, *P* = 0.000; for untreated: *F* = 4.89, *P* = 0.003). The capital letters indicate differences among the treatments (Treated or Un-Treated); lowercase letters indicates differences between treated and untreated surfaces, Tukey test (α = 0.05). Significant difference “***” and non-significant difference “ns”.

The *Panonychus citri* less visited the abaxial surface than the adaxial surface, so a small number of mites (Mean ± SE) were captured but enough for the difference between treated and untreated surfaces. At LC_30_ doses, the data collected from abaxial surface of right side (ABR) was significantly different on treated and untreated surfaces: Abamectin (*t*_−1.889_ = −6.8; *P* = 0.000), EnSpray 99 (*t*_−1.889_ = −3.3; *P* = 0.005) and control (*t*_−1.444_ = −2.25; *P* = 0.041). By treating ABR with lethal concentrations (LC_50_) was significantly different on treated and untreated surfaces by treated with vegetable oil (*t*_−3.222_ = −4.24; *P* = 0.002) while SYP-9625, abamectin, and EnSpray 99 were similar between the replication within treated or untreated. The number of mites found maximum on the un-treated ABR surface treated with LC_50_ of abamectin (6.78 ± 0.813) (Fig. 4).

**Figure 5:**
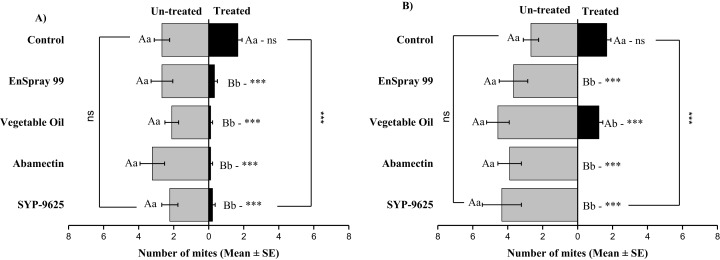
The number of *Panonychus citri* (Mean ± SE) re-captured after 24 h on the abaxial surface of leaves (left side); (A) LC_30_, (B) LC_50_. A significant difference was observed between treatments than control within treated surfaces (df = 4,14; For LC_30_: *F* = 15.01, *P* = 0.000; for LC_50_: *F* = 29.78, *P* = 0.000). The capital letters indicate differences among the treatments (Treated or Un-Treated); lowercase letters indicates differences between treated and untreated surfaces, Tukey test (α = 0.05). Significant difference “***” and non-significant difference “ns”.

The difference between treated and un-treated was observed significant within all treatments: SYP-9625 (*t*_−2_ = −4.1; *P* = 0.003), abamectin (*t*_−3.11_ = −4.37; *P* = 0.002), vegetable oil (*t*_−2_ = −4.94; *P* = 0.001) and EnSpray 99 (*t*_−2.33_ = −3.61; *P* = 0.006) except control at LC_30_ doses on the abaxial surface of left side (ABL). No mites were observed after treatment with LC_50_ doses on ABL except vegetable oil (*t*_−3.33_ = −4.87; *P* = 0.001) and control (non-significant). Maximum number of mites found on un-treated surfaces depending on the concentration of chemicals (Fig. 5).

**Figure 6:**
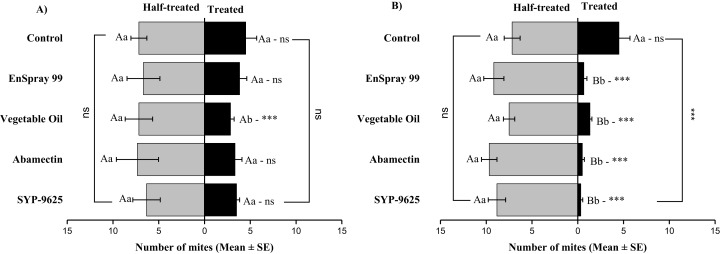
The number of *Panonychus citri* (Mean ± SE) re-captured after 24 h on the adaxial surface of leaves; (A) LC_30_, (B) LC_50_. A significant difference was observed between treatments than control within treated (df = 4,29; at LC_50_: *F* = 8.55, *P* = 0.000). The capital letters indicate differences among the treatments (Treated or Half-Treated); lowercase letters indicates differences between treated and half-treated surfaces, Tukey test (α = 0.05). Significant difference “***” and non-significant difference “ns”.

On the adaxial surfaces, difference between treated and half-treated surfaces were found similar (non-significant) at LC_30_ except on vegetable oil application (*t*_4.33_ = 2.8, *P* = 0.038) while at LC_50_, all treatments found significant different (For SYP-9625: *t*_8.5_ = 8.77, *P* = 0.000; abamectin: *t*_9.167_ = 10.51, *P* = 0.000; vegetable oil: *t*_6.167_ = 9.43, *P* = 0.000; EnSpray: *t*_8.5_ = 7.54, *P* = 0.001) (Fig. 6).

**Figure 7:**
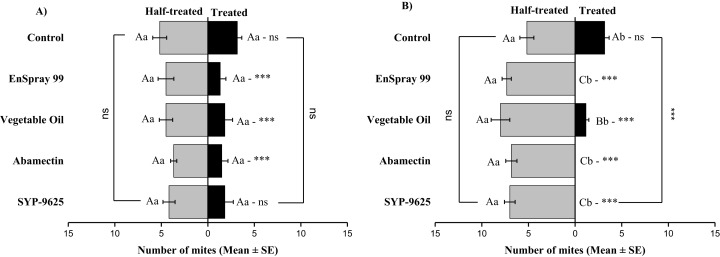
The number of *Panonychus citri* (Mean ± SE) re-captured after 24 h on the abaxial surface of leaves; (A) LC_30_, (B) LC_50_. A significant difference was observed between treatments than control within treated (df = 4,29; at LC_50_: *F* = 29.61, *P* = 0.000). The capital letters indicate differences among the treatments (Treated or Half-Treated); lowercase letters indicates differences between treated and half-treated surfaces, Tukey test (α = 0.05). Significant difference “***” and non-significant difference “ns”.

On the abaxial surfaces between treated and half-treated number of mites was significantly different at LC_30_: abamectin (*t*_2.167_ = 2.89; *P* = 0.023), vegetable oil (*t*_2.67_ = 2.42; *P* = 0.038) and EnSpray 99 (*t*_3.17_ = 3.03; *P* = 0.014) except SYP-9625 and control. At LC_50_, a significant difference was observed between treated and half-treated surfaces with all mites repelled from treated surfaces (SYP-9625, abamectin and EnSpray 99) (Fig. 7).

**Figure 8:**
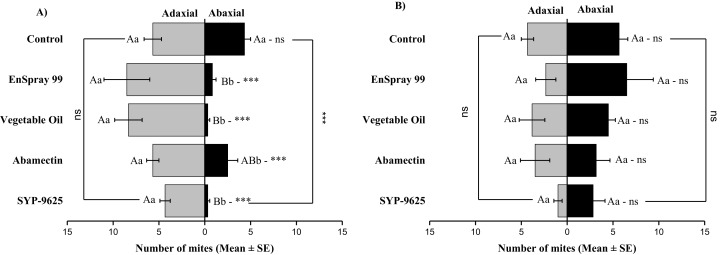
The number of *Panonychus citri* (Mean ± SE) re-captured after 24 h (the whole plant treated) on adaxial and abaxial surfaces. The results of LC30 (A) and LC50 (B) concentrations are presented. A significant difference was observed between treatments than control within abaxial (df = 4,29; at LC_30_: *F* = 8.32, *P* = 0.000). The capital letters indicate differences among the treatments (Adaxial or Abaxial surface); lowercase letters indicates differences between adaxial and abaxial surfaces, Tukey test (α = 0.05). Significant difference “***” and non-significant difference “ns”.

In no choice teste (whole plant treated), a significant difference was observed within all treatments (between adaxial and abaxial surfaces): SYP-9625 (*t*_−4_ = −6.71; *P* = 0.001), Abamectin (*t*_−3.17_ = −2.53; *P* = 0.035), vegetable oil (*t*_−8_ = −5.37; *P* = 0.003) and EnSpray 99 (*t*_−7.67_ = −3.04; *P* = 0.029) except control (*t*_−1.33_ = −1.15; *P* = 0.285) at LC_30_ doses while all treatments found no difference between adaxial and abaxial surfaces at LC_50_ doses (Fig. 8).

### Correlation analysis

The correlation between toxicity vs treated and toxicity vs un-treated on both surfaces, either right or left, were found negatively correlated except EnSpray and abamectin (Toxicity vs Treated) at LC_30_ and LC_50_, respectively (Table S1).

The relationship between toxicity and treated surfaces was positively correlated at LC_30_ on adaxial (SYP-9625, abamectin, and EnSpray) and abaxial surfaces (abamectin and EnSpray). There was a significant correlation between toxicity and sublethal half-treated abaxial surfaces of SYP-9625, abamectin, and EnSpray 99. There was a positive correlation between toxicity and lethal half-treated adaxial surface for vegetable oil and EnSpray 99. In contrast, on the abaxial surface, only SYP-9625 was found positively correlated (Table S2). In a no-choice experiment (the whole plant treated), a positive correlation was observed by treatment with vegetable oil (toxicity vs adaxial) at both concentrations (Table S3).

## Discussion

Mites disperse themselves by walking (Sabelis & Dicke, 1985) to find a suitable site for colonization and feeding (Tixier, Kreiter & Auger, 2000; Aguilar-Fenollosa et al., 2016; Moerman, 2016; Mukwevho, Olckers & Simelane, 2017; Sousa et al., 2019). One major factor for dispersal is environmental contamination, due to pesticide application (Lima et al., 2015; Guedes et al., 2016; Mohammed et al., 2019; Monteiro et al., 2019b). This study aimed to determine whether synthetic chemicals and oils respond similarly to the dispersal and colonization behavior of *Panonychus citri*. The physio-morphic characteristics of leaf such as leaf surfaces and leaf domatia play an essential role in habitat selection (O’Dowd & Pemberton, 1994, 1998; Tixier, Kreiter & Auger, 2000; English-Loeb, Norton & Walker, 2002; Romero & Benson, 2004). The majority of mites (Tetranychids) prefer to feed and oviposit on the leaves’ abaxial surface. In contrast, some phytophagous mites like *P. citri* and *Tetranychus urticae* Koch are preferred on both surfaces (Azandeme-Hounmalon et al., 2014). This mites distribution from treated surfaces due to chemical cues (Domingos et al., 2010; Melo et al., 2011) and maybe their phylogenetical responses (Rollo, Czyzewska & Borden, 1994; Nilsson & Bengtsson, 2004; Cisak et al., 2012; Buehlmann et al., 2014).

In the citrus growing region of South China, SYP-9625 and abamectin are commonly used against different pests, including citrus red mite (Meng, Wang & Jiang, 2002; Fang et al., 2013; Tian, Li & Ran, 2013; Liao et al., 2016; Dou et al., 2017). SYP-9625 is commonly used against phytophagous mites with minimum hazard to animals (Li et al., 2010; Chai et al., 2011; Tian, Li & Ran, 2013; Yu et al., 2016; Liu et al., 2018; Ouyang et al., 2018; Chen et al., 2019). Liu et al. (2018) reported that SYP-9625 gave maximum mortality and dispersed against *P. cirti* in the no-choice test, similar to our results and against *Tetranychus citri* (Chen et al., 2019). Abamectin showed less repellency than SYP-9625 against *P. citri* (Dou et al., 2017) due to resistance development (Hu et al., 2010; Liao et al., 2016).

By contrast to synthetic chemicals, plant-based derivatives (such as vegetable oils) are used as alternatives (Flamini, 2003) due to their compatibility with non-target organisms, low toxicity, negligible resistance development, and eco-friendly (Marcic, 2012). Fatty acids that are significant vegetable oil components are active ingredients that increase their toxicity against pests (Baldwin, Koehler & Pereira, 2009; Sims et al., 2014). Linoleic acid that is an important component of vegetable oil resulted in attractive responses (Rollo, Czyzewska & Borden, 1994; Buehlmann et al., 2014), as *P. citri* found on treated surfaces (at LC_50_) after 24 h in this study. The short-chain compound (palmitic acid) in vegetable oil gave equal repellency to synthetic chemicals in previous studies (Mullens, Reifenrath & Butler, 2009; Buehlmann et al., 2014). Vegetable oils gave similar responses to synthetic chemicals with a slow mode of action. They can be used as an alternative against *P. citri* with Ribeiro et al. (2014) endorsement.

EnSpray 99 exhibits minimum toxic residues on the treated fruit surfaces by losing their toxicity (Zhuang et al., 2015). The efficacy of EnSpray 99 has been reported against different pests, including citrus red mites by many researchers (Wang, Gu & Bei, 2004; Chen & Zhan, 2007; Li & Zhang, 2011; Zhou et al., 2011; Zhuang et al., 2015). The EnSpray 99 contains paraffinic oil more than 60%, which was also found on the fruit residues (Ahmad et al., 2018) and effectively used against *P. citri* (Riehl & Jeppson, 1953; Trammel, 1965). The study shows that EnSpray 99 responded similarly to vegetable oil and synthetic chemicals against the repellency and dispersal of *P. citri*. The recommended concentrations ranging from 0.5% to 1.4% against *P. cirti* and eriophyids (Benfatto et al., 2002; Tang et al., 2002) while Wang, Gu & Bei (2004) used 14.11 mgL^−1^ (LC_50_) against *P. citri* in the laboratory. EnSpray 99 can be used against *P. citri* control strategies by keeping their impact on pest resistance development, environmental contamination, plant growth reduction, and chronic and acute effect on humans (Ahmed & Fakhruddin, 2018).

According to a free-choice bioassay on dispersal, all mites were significantly dispersed towards the un-treated and half-treated surfaces. According to Alves, Casarin & Omoto (2005), untreated surfaces were significantly preferred by the *P. citri* at the adult stage for feeding and oviposition. Maximum dispersal from treated to un-treated or half-treated surfaces depended on the concentration of chemicals Iftner & Hall (1983). The dispersal towards half-treated adaxial surfaces was significantly different from vegetable oil application than others at LC_30,_ as observed by Alves, Casarin & Omoto (2018).

The comprehensive assessments of these chemicals against *P. citri* need a more detailed study. The surface treated with these chemicals may affect natural enemies efficiency. However, the experiment carried out here did not evaluate the other factors and needed attention to more applied work.

## Conclusions

In conclusion, *P. citri* preferred site adaxial surfaces of citrus leaves for feeding and colonization which were the best sprayed sites for acaricides. However, spraying more times and unequally, *P. citri* would disperse more quickly. Vegetable oil and EnSpray 99 were the least affecting the colonization depending on mite release point and SYP-9625 gave maximum repellency with a higher number of missing or dead mites recorded.

## Supplemental Information

10.7717/peerj.10899/supp-1 Supplemental Information 1 Correlation between toxicity, treated and untreated leaf surfaces by chemicals on *P. citri*. C.I. is the confidence interval for r (correlation co-efficient).

10.7717/peerj.10899/supp-2 Supplemental Information 2 Correlation between toxicity, treated and half-treated by chemicals on *P. citri*. Significant correlation in bold. C.I. is the confidence interval for r (correlation coefficient).

10.7717/peerj.10899/supp-3 Supplemental Information 3 Correlation between toxicity, adaxial and abaxial surface, fully treated with chemicals on *P. citri*. Significant correlation in bold. C.I. is the confidence interval for r (correlation coefficient).

10.7717/peerj.10899/supp-4 Supplemental Information 4 Raw data. Includes LC_30_, LC_50_ and toxicity raw result.

## Additional Information and Declarations

### Data Availability

The following information was supplied regarding data availability: Raw data are available in the Supplemental Files.
